# Comprehensive Expression Profiling and Functional Network Analysis of Porphyra-334, One Mycosporine-Like Amino Acid (MAA), in Human Keratinocyte Exposed with UV-radiation

**DOI:** 10.3390/md15070196

**Published:** 2017-06-24

**Authors:** Sung-Suk Suh, Sung Gu Lee, Ui Joung Youn, Se Jong Han, Il-Chan Kim, Sanghee Kim

**Affiliations:** 1 Division of Polar Life Sciences, Korea Polar Research Institute, Incheon 21990, Korea; sung-suk.suh@kopri.re.kr (S.-S.S.); holynine@kopri.re.kr (S.G.L.); ujyoun@kopri.re.kr (U.J.Y.); hansj@kopri.re.kr (S.J.H.); 2 Department of Polar Science, University of Science and Technology, Incheon 21990, Korea

**Keywords:** Mycosporine-like amino acids (MAAs), UV-radiation, porphyra-334, microRNAs, Wnt pathway, Notch pathway

## Abstract

Mycosporine-like amino acids (MAAs) have been highlighted as pharmacologically active secondary compounds to protect cells from harmful UV-radiation by absorbing its energy. Previous studies have mostly focused on characterizing their physiological properties such as antioxidant activity and osmotic regulation. However, molecular mechanisms underlying their UV-protective capability have not yet been revealed. In the present study, we investigated the expression profiling of porphyra-334-modulated genes or microRNA (miRNAs) in response to UV-exposure and their functional networks, using cDNA and miRNAs microarray. Based on our data, we showed that porphyra-334-regulated genes play essential roles in UV-affected biological processes such as Wnt (Wingless/integrase-1) and Notch pathways which exhibit antagonistic relationship in various biological processes; the UV-repressed genes were in the Wnt signaling pathway, while the activated genes were in the Notch signaling. In addition, porphyra-334-regulated miRNAs can target many genes related with UV-mediated biological processes such as apoptosis, cell proliferation and translational elongation. Notably, we observed that functional roles of the target genes for up-regulated miRNAs are inversely correlated with those for down-regulated miRNAs; the former genes promote apoptosis and translational elongation, whereas the latter function as inhibitors in these processes. Taken together, these data suggest that porphyra-334 protects cells from harmful UV radiation through the comprehensive modulation of expression patterns of genes involved in UV-mediated biological processes, and that provide a new insight to understand its functional molecular networks.

## 1. Introduction

Under exposure of environmental ultraviolet (UV) radiation, certain living organisms have developed characteristic defense mechanisms to diminish the adverse effects of UV radiation, including DNA damage and the production of reactive oxygen species (ROS) [1,2,3]. Recent studies have reported that many photosynthetic marine organisms can synthesize secondary metabolites with UV-absorbing capacity such as mycosporine-like amino acids (MAAs), as one of the most effective UV protection mechanisms [4,5]. These compounds have characteristic chemical structure with either an aminocyclohexenone or an aminocycloheximine ring, which provides them with ability to absorb UV radiation and be resilient to DNA damage by harmful UV radiation. MAAs have been found in a wide range of marine organism exposed to environmental UV radiation, and the chemical structures of more than 30 different MAAs have been characterized [6,7,8,9]. Although a number of recent reports for MAAs in various marine organisms refers mostly to their UV protective ability, scientific evidence is increasingly accumulating that MAAs contribute other functional roles such as antioxidant activity and osmotic regulation. For example, porphyra-334, which has characteristic chemical structure for UV absorption (Supplementary Materials, Figure S1), exerts potent antioxidant activity and prevents cellular damage caused by UV-induced ROS with free radical scavenging capacity [10,11,12]. This fact suggests that MAAs can play crucial roles as antioxidant molecules to modulate cellular processes affected by ROS, such as DNA damage and apoptosis. In addition to antioxidant activity of MAAs, these compounds could regulate osmotic pressure within the cell [13]. To maintain the essential osmotic balance, most organisms accumulate small weight molecules without charge into cells, which can function as so-called “osmotic solutes”. In this aspect, MAAs, which are small uncharged organic molecules, can contribute to intracellular osmotic pressure and provide organism with capability to adapt to extreme environments with high salt concentrations. Despite abundant ecological and physiological studies available on the functional roles of MAAs, our understanding of their roles at the molecular level remains poor. Recently, molecular biological studies on the roles of MAAs have been just begun. For example, MAAs from green algae protect skin against UV-induced skin damage through recovery of UV-suppressed expression of skin aging-related genes [14,15,16,17,18,19]. In addition, they function as effective drugs on immunomodulatory effects [20] and the wound healing process in human keratinocytes through the activation of the various genes, such as focal adhesion kinases (FAK), extracellular signal-regulated kinases (ERK), and c-Jun *N*-terminal kinases (JNK) [21]. Nevertheless, there is a need for extensive molecular mechanism studies to explore the photoprotective roles of MAAs so that a range of industrial and pharmaceutical applications can be found. Many studies have focused on marine organisms as a source of natural bioactive molecules having a photoprotective role, their biosynthesis and commercial application [4,5]. In this aspect, MAAs have attracted considerable research attention in both industrial and pharmacological fields. Recently, we have demonstrated that the expression of skin aging-related genes such as procollagen C proteinase enhancer (PCOLCE), elastin and involucrin were modulated by porphyra-334 in a dose dependent manner [11], and porphyra-334 significantly attenuates UV-induced apoptosis in HaCaT cells through the activation of caspase pathway [22]. In the present study, we firstly investigated the comprehensive molecular networks that are associated with the functional roles of porphyra-334 as an UV-absorbing substance in human keratinocyte.

## 2. Results and Discussion

### 2.1. Porphyra-334-Modulated Differentially Expressed Genes (DEGs)

**Figure 1:**
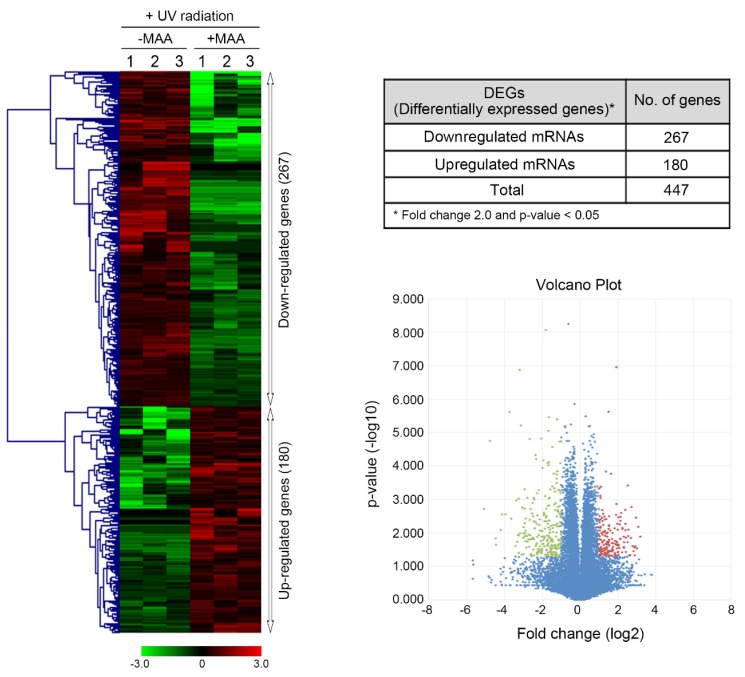
Gene expression profiling in response to porphyra-334 treatment in UV-exposed human keratinocyte. Differentially expressed genes (*p* < 0.05) were analyzed by hierarchical clustering of the log 2 value of each RNA microarray signal. Red: up-regulation; green: down-regulation; black: no change. In the volcano plot for differential gene expression, red dots indicate significantly up-regulated genes, while green dots indicate downregulated genes. Blue dots represent no significant genes.

**Figure 2:**
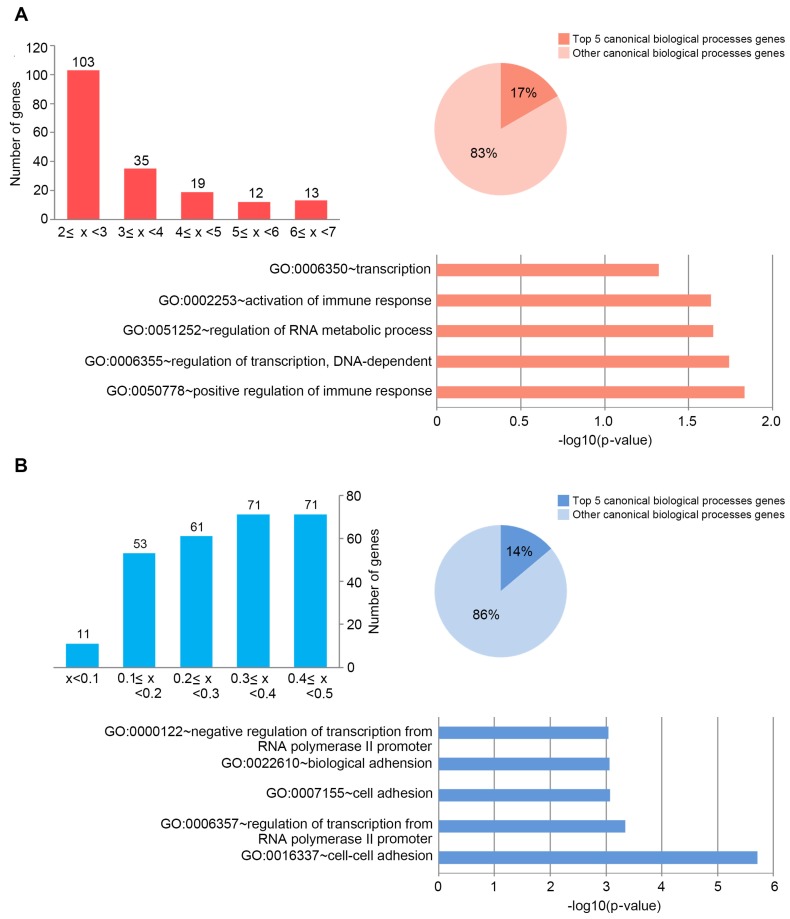
Distribution of gene expression and gene ontology analysis of: porphyra-334-up-regulated genes (**A**); and porphyra-334-down-regulated genes (**B**). Bar charts showing the distribution of expression of: up-regulated genes (**A**); and down-regulated genes (**B**) in porphyra-334 treated cells exposed to UV radiation. Pie charts showing overlap of: up-regulated genes (**A**); and down-regulated genes (**B**) involved in top five or other canonical biological processes. The chart fragments represent the number of genes associated with the various terms. The gene set induced by each corticosteroid was functionally classified according to Gene Ontology terms.

Gene expression profiling by high throughput sequencing was performed to investigate the effect of porphyra-334 on UV-modulated transcriptome in UV-exposed HaCaT cells. We focused on the identification of genes whose expression was significantly altered in the porphyra-334 treated group compared with the non-treated group (*p* value < 0.05). The application of this threshold led to the identification of 447 DEGs, of which 267 were over-expressed and 180 were under-expressed in porphyra-334 treated group (Figure 1). The largest number of up- and down-regulated genes, 103 and 71, exhibited about two-fold increase in their transcriptional levels (2 ≤ x < 3) or less than threefold decrease (0.3 ≤ x < 0.4 or 0.4 ≤ x < 0.5), respectively (Figure 2). To gain a better understanding about their biological function, gene set enrichment analysis was performed to identify significantly over- and under-represented gene ontology (GO) categories (Supplementary Materials, Tables S1 and S2, respectively). Approximately 17% of up-regulated genes in porphyra-334 treated cells were in the top five canonical biological processes (Figure 2B) which were strongly associated with the immune response, regulation of transcription and RNA metabolic process (Figure 2A). Genes highly activated in response to the treatment of porphyra-334, including *Pla2g7*, *Pitx2*, *Gpr34* and *Fbf1* genes, are specifically expressed in the immune response or Wnt signaling pathway [22,23,24,25] which is necessary for proper development and regeneration of various tissues including bone, heart and muscle. In addition, it has been known that this pathway was clinically important because its dysregulation can lead to various diseases, including breast, prostate, glioblastoma, and diabetes [26,27,28,29]. The expression of these genes was verified by qRT-PCR analysis (Supplementary Materials, Figure S2). In contrast, approximately 14% of the total down-regulated genes in porphyra-334 treated cells involved in the top five biological process (Figure 2A) which were strongly related to cell-cell adhesion, biological adhesion and regulation of transcription (Figure 2B). Among them, the significantly down-regulated genes, such as *Rasd1*, *Fgf12*, *Nkx2-5* and *Cpeb1*, were involved in these categories. Notably, they are directly or indirectly involved in Notch signaling pathway [30,31,32,33,34,35], which is evolutionarily conserved and responsible for cell fate determination in the developing embryo and mature tissue in a highly tissue context- and cell- type-dependent manner. For example, Rasd1 has been shown to interact with EAR2 [30], the orphan nuclear receptor, which can activate Notch signaling [31,32]. In addition, Notch signaling can be promoted downstream of FGF in developmental processes such as stem cells [33], suggesting that FGF12, a component in the FGF signaling pathways, can be involved in Notch signaling. Many studies demonstrate that Notch signaling increases tumor cell proliferation and is activated in the cancer stem-cell pool [36,37]. Thus, pharmacological inhibition of Notch pathway can improve the effectiveness of cancer treatment and patient survival. Recently, many studies report that Wnt and Notch pathways interact either in synergistic or antagonistic manners to exert their biological roles [36,37].

For example, Notch and Wnt signals play essential roles in cell proliferation and tumorigenesis through the synergetic effects between Notch and Wnt signaling; the dramatic proliferative effect was observed when Notch and Wnt signals are normal in the intestinal development [38]. In contrast, in response to UV-radiation, keratinocyte-derived Wnt signaling inhibits Notch signaling through the activation of the Notch inhibitor such as Numb via Wnt pathway-dependent manner [39]. In addition, Notch-dependent suppression of *Pitx2* gene transcription, which consists in Wnt signaling, was demonstrated in cardiac/laterality defects [40]. These observations are consistent with our results that, at least in part, the expression of porphyra-334-regulated genes could be controlled by Wnt or Notch signaling in antagonistic manners in UV-exposed keratinocyte. Our data show that the biological antagonism of the Wnt/Notch signaling pathways may be relevant to the homeostasis of keratinocyte in the human skin microenvironment against the harmful UV-radiation, implying that the functional disturbance between two pathways may contribute to cause cell damage which may potentially induce skin carcinogenesis. In fact, it has been demonstrated that Notch signaling is frequently dysregulated, most commonly by over-activation, across many cancers including melanoma [41]. According to our data, porphyra-334 considerably decreased the expression level of genes related with Notch signaling. This observation suggests that, at least in part, porphyra-334 can inhibit progression of skin carcinogenesis against UV-radiation. In contrast, it has been known that the inhibition of Notch pathway impairs cell growth and induces apoptosis in UV-exposed cells [38,42]. Thus, the significant suppression of Notch-associated genes in UV-exposed HaCaT cells may induce apoptosis or cell death, even in the presence of UV-absorbing compound porphyra-334, implying that porphyra-334 cannot completely protect from the harmful effect of UV-radiation.

### 2.2. Porphyra-334-Modulated miRNAs

MicroRNAs (miRNAs), small noncoding RNAs of ~22 nts that mediate posttranscriptional silencing of specific target mRNAs, are increasingly being recognized as an important determinant in a variety of biological processes [43]. Deregulated miRNAs were suggested to exert their function in organism through silencing of key cell fate regulators by directly binding their 3′ UTR [44]. Recently, many studies have been demonstrated that miRNAs play a critical role in molecular regulatory mechanism of UV-induced cellular processes such as DNA damage, apoptosis, deregulation of cell cycle and generation of ROS [45]. To determine the effect of porphyra-334 on the expression pattern of UV-affected miRNAs, we used the nanoString nCounter platform for profiling expression of miRNAs in the porphyra-334 treated cells and control cells; miRNAs up- or down-regulated by >1.5-fold change and an expression level higher than 100 code counts were further analyzed.

**Figure 3:**
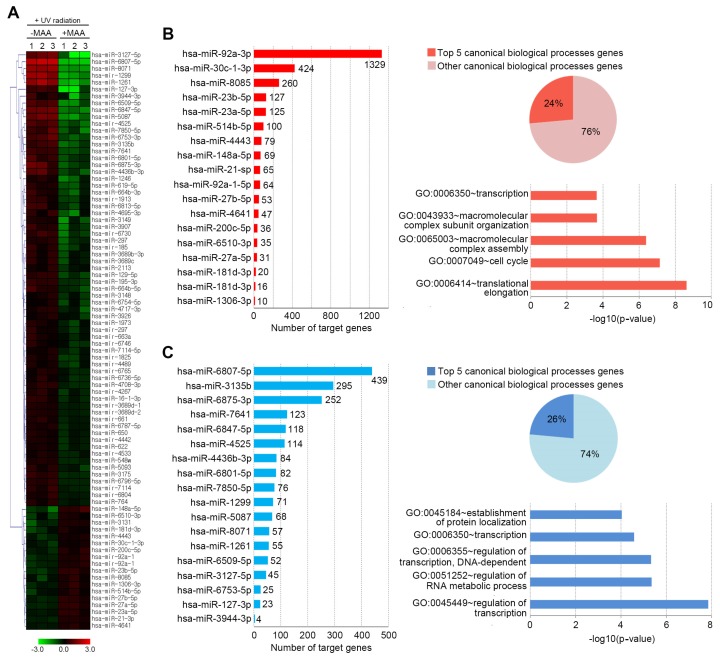
A profiling of microRNA expression and gene ontology analysis in response to porphyra-334 treatment in UV-exposed human keratinocyte. Differentially expressed miRNAs (*p* < 0.05) were analyzed by hierarchical clustering of the log 2 value of each RNA microarray signal (**A**). Bar charts showing the number of target genes for: up-regulated miRNAs (**B**); and down-regulated miRNAs (**C**). Pie charts showing overlap of candidate target genes for: up-regulated miRNAs (**B**); and down-regulated miRNAs (**C**), which were involved in top five or other canonical biological processes. The chart fragments represent the number of target genes associated with the various terms.

Unexpectedly, widespread repression of miRNAs expression in this model system was observed in response to porphyra-334 treatment (66 p53-repressed miRNAs of 84 total p53-responsive miRNAs; *P* < 0.05) (Figure 3A). We identified differentially expressed 84 miRNAs (DEmiRNAs) when comparing porphyra-334 treated group and control (Figure 3A), of which 18 were over-expressed and 66 were under-expressed in the porphyra-334 treated group. To further characterize the porphyra-334-regulated miRNAs in UV-exposed cells, we focused on the most robustly-changed miRNAs; among the DEmiRNAs, the highest ranked 18 up- or down-regulated miRNAs stood out as attractive candidates for a role in porphyra-334-related function. Consistent with previous studies [46,47], among the up-regulated miRNAs, hsa-miR-92a-3p, -21-3p and -27a-5p were identified as UV-induced miRNAs in human keratinocyte. For example, has-miR-92a-3p and -27a-5p were highly expressed in UV-exposed human dermal papilla cells, while hsa-miR-21 was activated in mouse fibroblast upon UV exposure. In addition, some up-regulated miRNAs such as hsa-miR-30c, -23a, -148a, -200c, and -181d have been identified as p53-regulated miRNAs [48,49]. In general, p53 can play a key role in response to DNA damage caused by various types of cellular stresses including UV-radiation [50]. In fact, we observed that UV-elevated level of p53 mRNAs was significantly decreased by porphyra-334 (Supplementary Materials, Figure S3). Therefore, it can be supposed that the above miRNAs are involved in UV-mediated cellular functions in human keratinocyte. Next, to gain insight into the biological functions of 18 porphyra-334-regulated DEmiRNAs, we predicted miRNA target genes using widely accepted miRNA-target-predicting software PicTar and TargetScan 5.1 algorithms which are the most popular miRNA target gene prediction tools. To reduce false positives, target genes represented in both databases were chosen for further analyses. Finally, the number of total putative target genes of the 18 up-regulated miRNAs was 2890 genes, while the down-regulated miRNAs had 1983 genes. Notably, hsa-miR-92a-3p (among the up-regulated miRNAs) and hsa-miR-6807-5p (among the down-regulated miRNAs) had the most target genes, 1329 and 439, respectively (Figure 3). The expression of both miRNAs was verified by qRT-PCR analysis (Supplementary Materials, Figure S3).

**Figure 4:**
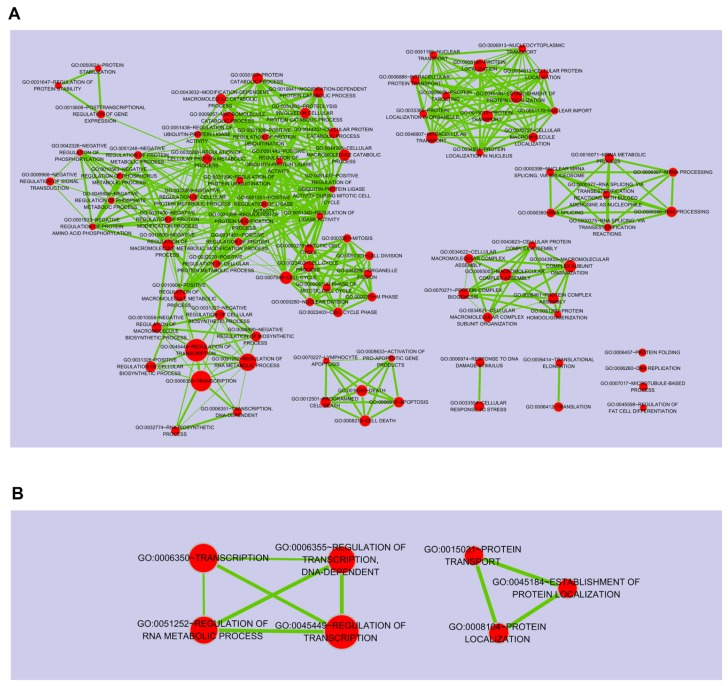
Gene set enrichment analysis (GSEA) of the predicted target genes for up-regulated miRNAs (**A**); and down-regulated miRNAs (**B**). The node size indicates the significance of the enrichment.

To gain a better understanding of the functional implications of these miRNAs, we performed enrichment analysis for their putative target genes using the DAVID tools (Supplementary Materials, Table S3). Approximately 24% of putative target genes for the up-regulated miRNAs were strongly associated with the transcription, macromolecular complex subunit organization, macromolecular complex assembly, cell cycle and translational elongation (Figure 3B). In contrast, about 26% of putative target genes for the down-regulated miRNAs were mainly related to protein localization, regulation of transcription, and regulation of RNA metabolic process (Figure 3C). The full list of significant GO terms is given in Supplementary Materials, Table S4. Next, to examine the biological pathways enriched in the data set in an unbiased, systematic method, gene set enrichment analysis (GSEA) was performed. This software examines gene expression data at the level of gene sets, which are based on existing biological pathway or co-expression data from published research within the Molecular Signature Database [51]. GSEA results were applied to Enrichment Map in Cytoscape to generate a large network of enriched gene sets, and then the large network was clustered to generate sub-networks of interrelated gene sets (Figure 4).

### 2.3. Target DEGs were Inversely Correlated with Functionally Enriched miRNAs

**Figure 5:**
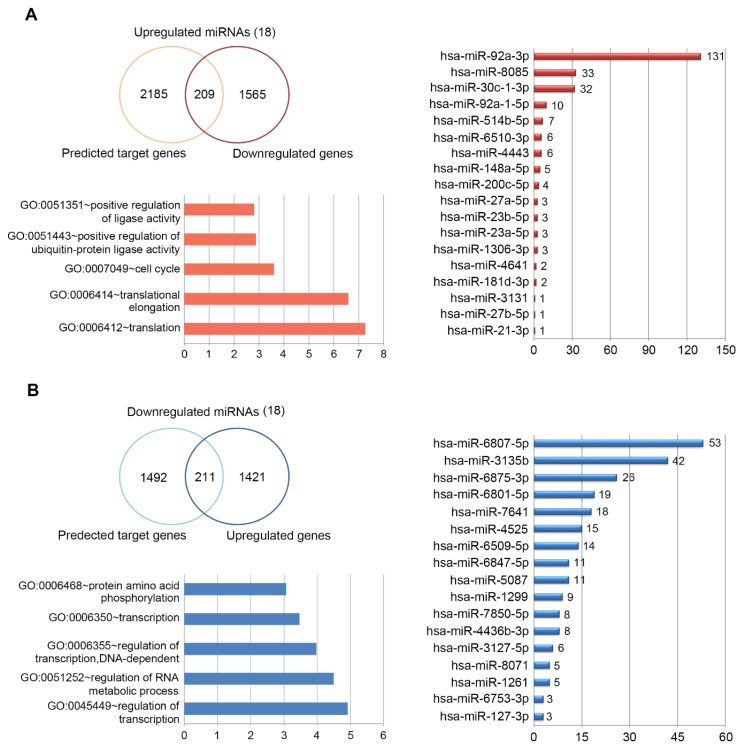
Distribution and top five enriched gene ontology terms of candidate target genes for the: up-regulated miRNAs (**A**); and down-regulated miRNAs (**B**).

**Figure 6:**
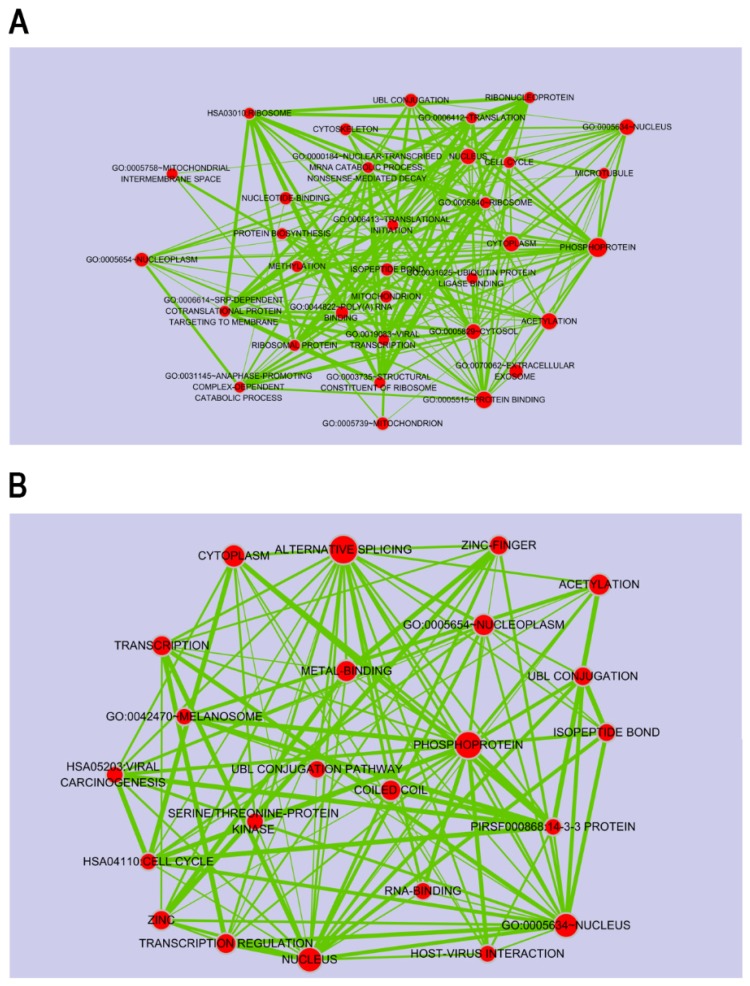
Gene set enrichment analysis (GSEA) of the candidate target genes for up-regulated miRNAs (**A**); and down-regulated miRNAs (**B**). The node size indicates the significance of the enrichment.

Next, to further understand the role of DEmiRNAs, we searched for their putative target genes from DEGs profiling data. In the DEGs data, genes up- or down-regulated by >1.2-fold change were selected for further analysis because miRNAs can decrease the mRNA levels of target genes at the different degree. For example, despite a >95% decrease in protein expression, only small decrease (approximate 20% or less than) in mRNA abundance was observed [52]. Therefore, genes with fold change above 1.2 were considered as their putative target genes. Application of this threshold led to the identification of 3406 DEGs, of which 1774 were under-expressed (Figure 5A) and 1632 were over-expressed (Figure 5B). To build a comprehensive list of all the putative validated target genes, we compared them with the predicted target genes using miRNA prediction programs such as TargetScan and Pictar software. In total, these DEmiRNAs regulated 4097 putative target genes against 18 up- or down-regulated miRNAs. We looked at whether the DEGs that were significantly and negatively correlated with DEmiRNAs were in fact miRNA targets; of which 2394 genes were on the predicted target genes for the up-regulated miRNAs (Figure 5A), while 1703 genes were for down-regulated miRNAs (Figure 5B). Ultimately, we considered a total of 209 (for 18 up-regulated miRNAs) (Figure 5A) and 211 (for 18 down-regulated miRNAs) (Figure 5B) enriched RNAs, showing that their expression patterns were inversely correlated with DEmiRNAs in response to porphyra-334. Among the DEmiRNAs, has-miR-92a-3p and -6807-5p have the most number of target genes, 131 and 53, respectively (Figure 5). To identify the most relevant cellular activities controlled by each anti-correlated target DEGs, we analyzed overrepresented GO biological process terms. The most significant GO terms for target genes of up-regulated miRNAs were related to regulation of ligase activity, regulation of ubiquitin-protein ligase activity, cell cycle, translational elongation and translation (Figure 5A; Supplementary Materials, Table S4). In contrast, in the case of target genes of down-regulated miRNAs, they were discovered to participate in biological processes associated with protein amino acid phosphorylation, transcription, DNA-dependent regulation of transcription, regulation of RNA metabolic process and regulation of transcription (Figure 5B; Supplementary Materials, Table S5). Notably, between two different groups of target DEGs, we found that there is inverse correlation with respect to UV-related biological processes including apoptosis, cell death, cell cycle, and regulation of cell proliferation; target genes for the up-regulated miRNAs are involved in apoptosis, mitotic cell cycle and translational elongation, whereas those for the down-regulated miRNAs function as anti-apoptosis, anti-cell death and positive regulators of cell proliferation. In the previous study [53], following UV DNA damage, there is an overall inhibition of protein synthesis and translational reprogramming. This reprogramming allows the cells to repair DNA damage and counter with apoptosis through the selective synthesis of specific proteins including elongation factors and DDR proteins. Next, to examine the biological pathways enriched in the data set in an unbiased, systematic method, GSEA was performed to generate a large network of enriched gene sets, and then the large network was clustered to generate sub-networks of interrelated gene sets (Figure 6). Taken together, these data suggest that porphyra-334 may protect the cells from harmful UV-radiation through the modulation of genes associated with cell survival processes such as apoptosis, cell death and translational elongation.

## 3. Experimental Section

### 3.1. Cell Cultures and Irradiation

HaCaT cells were maintained in Dulbecco’s modified Eagle’s medium (DMEM; Gibco/Invitrogen, Thermo Fisher Scientific, Waltham, MA, USA) with 10% Fetal Bovine Serum (FBS) in 37°C humidified incubator containing 5% CO_2_. Cells were plated at a density of 0.5 × 10^6^/well in 6-well plates and grown overnight. They were incubated with porphyra-334 (1.0 mg/mL) for 30 min prior to UV irradiation, which was carried out as described previously [11]. Briefly, a Philips Original Home Solarium sun lamp (model HB 406/A; Philips, Grogningen, Holland) equipped with a UV lamp delivering a flux of 23 mW/cm^2^ between 300 and 400 nm was used as a UV radiation source. Cells were irradiated for 15 min (275 kJ/m^2^). This UV dose is equivalent to ~90 min of sunshine on the French Riviera (Nice, French) in the summer at noon [54].

### 3.2. RNA extraction

Total RNA was extracted using TRIzol Reagent (Invitrogen, Carlsbad, CA, USA) following the manufacture's instruction. Specifically, the pellet obtained from 5 × 10^6^ cells was lysed 1 mL of TRIzol solution. At the end of the extraction the isolated RNA was dissolved in 35 µL in RNase-free water and incubated for 10 min at 55 °C. An aliquot of 5 µg RNA was then used for cDNA synthesis using the SuperScript first strand cDNA synthesis kit (Invitrogen).

### 3.3. MicroRNA Microarray Analysis

For control and test RNAs, the synthesis of target miRNA probes and hybridization were performed using Agilent’s miRNA Labeling Reagent and Hybridization kit (Agilent Technologies, Palo Alto, CA, USA) according to the manufacturer’s instructions. Briefly, each 100 ng of total RNA were dephosphorylated with ~15 Units of calf intestine alkaline phosphatase (CIP), followed by RNA denaturation with ~40% DMSO and 10 min incubation at 100 °C. Dephosphorylated RNA were ligated with pCp-Cy3 mononucleotide and purified with MicroBioSpin 6 columns (Bio-Rad, Hercules, CA, USA). After purification, labeled samples were re-suspended with Gene Expression blocking Reagent and Hi-RPM Hybridization buffer, followed by boiling for 5 min at 100 °C and 5 min chilled on ice. Finally, denatured labeled probes were pipetted onto assembled Agilent Human miRNA Microarray (Human miRNA Microarray Release XX, AXBK) and hybridized for 20 hours at 55 °C with 20 RPM rotating in Agilent Hybridization oven (Agilent Technologies, Santa Clara, CA, USA). The hybridized microarrays were washed as the manufacturer’s washing protocol (Agilent Technologies).

### 3.4. Data Acquisition and Analysis

The hybridized images were scanned using Agilent’s DNA microarray scanner and quantified with Feature Extraction Software (Agilent Technologies). All data normalization and selection of fold-changed probes were performed using GeneSpringGX 7.3 (Agilent Technologies). We performed data transformation (set measurements less than 0.01 to 0.01) and per chip (normalize to 75th percentile) normalization. Probes which were changed not less than 2.0-fold of ratio between test and control samples were selected and considered as differentially expressed probes.

### 3.5. Library Oreparation and Sequencing

For control and test RNAs, the construction of library was performed using SENSE mRNA-Seq Library Prep Kit (Lexogen Inc., Vienna, Austria) according to the manufacturer’s instructions. Briefly, each 2 µg total RNA are prepared and incubated with magnetic beads decorated with oligo-dT and then other RNAs except mRNA was removed by washing solution. Library production is initiated by the random hybridization of starter/stopper heterodimers to the poly (**A**) RNA still bound to the magnetic beads. These starter/stopper heterodimers contain Illumina-compatible linker sequences. A single-tube reverse transcription and ligation reaction extends the starter to the next hybridized heterodimer, where the newly-synthesized cDNA insert is ligated to the stopper. Second strand synthesis is performed to release the library from the beads, and the library is then amplified. Barcodes were introduced when the library is amplified. High-throughput sequencing was performed as paired-end 100 sequencing using HiSeq 2000 (Illumina, Inc., San Diego, CA, USA).

### 3.6. Data Analysis

mRNA-Seq reads were mapped using TopHat software tool (https://ccb.jhu.edu/software/tophat) in order to obtain bam file (alignment file). Read counts mapped on transcripts region were extracted from the alignment file using bedtools (v2.25.0, http://bedtools.readthedocs.io/en/stable/) and Bioconductor that uses R (version 3.2.2, https://cran.r-project.org/bin/windows/base/old/3.2.2/) statistical programming language (R development Core Team, 2011). The alignment file also was used for assembling transcripts, estimating their abundances and detecting differential expression of genes or isoforms using cufflinks. We used the FPKM (fragments per kilobase of exon per million fragments) as the method of determining the expression level of the gene regions. Global normalization method was used for comparison between samples. Functional gene classification was performed by DAVID (http://david.abcc.ncifcrf.gov/). Cytoscape (version 2.7, http://www.cytoscape.org), an open source bioinformatics platform, provided by the U.S. National Institute of General Medical Sciences (NIGMS) (https://www.nigms.nih.gov) was used to construct network diagrams.

### 3.7. qRT-PCR Analysis

First-strand cDNAs were synthesized from 1 μg of total RNA using a high capacity cDNA reverse transcription kit (ThermoFisher Inc), and the samples were analyzed with sybr green real-time PCR master mixes (ThermoFisher Inc) with gene expression primers. The oligonucleotide primers used were as follows: p53, 5’-CCACCATCCACTACAACTACAT-5’ and 5’-AGGACAGGCACAAACACG-5’; Nkx2-5, 5’-CTCCCAACATGACCCTGAGT-3’ and 5’-CTCATTGCACGCTGCATAAT-3’; CPEB1, 5’-GTGATCCCCTGGGTATTAGC-3’ and 5’-CAGATATGACACAGAGAATCTT-3’; FGF12, 5’-AGCTTGGTTTCTGGGACTCA-3’ and 5’-CTCTTCTGAGATGAGTTTCTGCTC-3’; GPR34, 5’-GGGACTGGTTGGGAACATAA-3’ and 5’-GAAAGGGAGGCAGAAGATGA-3’; Pitx2, 5’-TCGTCCATGAACTGCATGAAAG-3’ and 5’-ATGTCATCGTAGGGCTGCATG-3’; Pla2g7, 5’-TAATGATCGCCTTGACACCCT-3’ and 5’-TACAGCAGCAACTATAAACCC-3’; Fbf1, 5’-TGTCAGCTCGGTATCTGTCG-3’ and 5’-GTGGTCTCTGCGCATGTCTA-3’; Rasd1, 5’-GCGGCGAAGTCTACCAGTTG-3’ and 5’-TGTCTAAGCTGAACACCAGAATGA-3’; GAPDH, 5’-GAAGGTGAAGGTCGGAGTC-3’ and 5’-GAAGATGGTGATGGATTTC-3’. For miRNAs, qRT-PCR primers for has-miR-92a-3p (product no: 204258) and has-miR-6807-5p (product no: 2107829) were purchased from Exiqon (Vedbæk, Denmark). As a control, U6 snRNA PCR primer (product no: 308006) was used (Exiqon).

## 4. Conclusions

In the present study, we showed that porphyra-334-regulated genes were involved in UV-affected biological processes such as Wnt and Notch pathways. In addition, porphyra-334-regulated miRNAs can also target a variety of genes associated with UV-affected biological processes such as apoptosis, cell proliferation and translational elongation. Notably, we observed good inverse correlation respect to biological functions between two different target groups for up- or down-regulated miRNAs. Taken together, these comprehensive molecular data indicate that porphyra-334 can be mostly involved in UV-affected biological pathways to protect organisms from harmful UV radiation, and that provide a new insight to understand its functional molecular networks.

## Supplementary Materials

The following are available online at www.mdpi.com/1660-3397/15/7/196/s1.
