# Supplementary material for: Comprehensive Expression Profiling and Functional Network Analysis of Porphyra-334, One Mycosporine-Like Amino Acid (MAA), in Human Keratinocyte Exposed with UV-radiation
Source: Mar Drugs. 2017 Jun 24;15(7):196. doi: 10.3390/md15070196 (PMC5532638; doi:10.3390/md15070196)
Supplement: Supplementary file 1 [file marinedrugs-15-00196-s001.zip › Supplementary Materials.pdf]

Legends of supplementary figures

Supplementary figure 1. A chemical structure of porphyra-334

Supplementary figure 2. Relative expression levels of highly up- or downregulated genes in Porphyra-334-modulated differentially expressed genes (DEGs) by quantitative RT-PCR

Supplementary figure3. Relative expression levels of p53 mRNAs (A) and porphyra-334-responsive miRNAs (B) by quantitative RT-PCR
