# Supplementary figures and images for: Comprehensive Expression Profiling and Functional Network Analysis of Porphyra-334, One Mycosporine-Like Amino Acid (MAA), in Human Keratinocyte Exposed with UV-radiation
Source: Mar Drugs. 2017 Jun 24;15(7):196. doi: 10.3390/md15070196 (PMC5532638; doi:10.3390/md15070196)

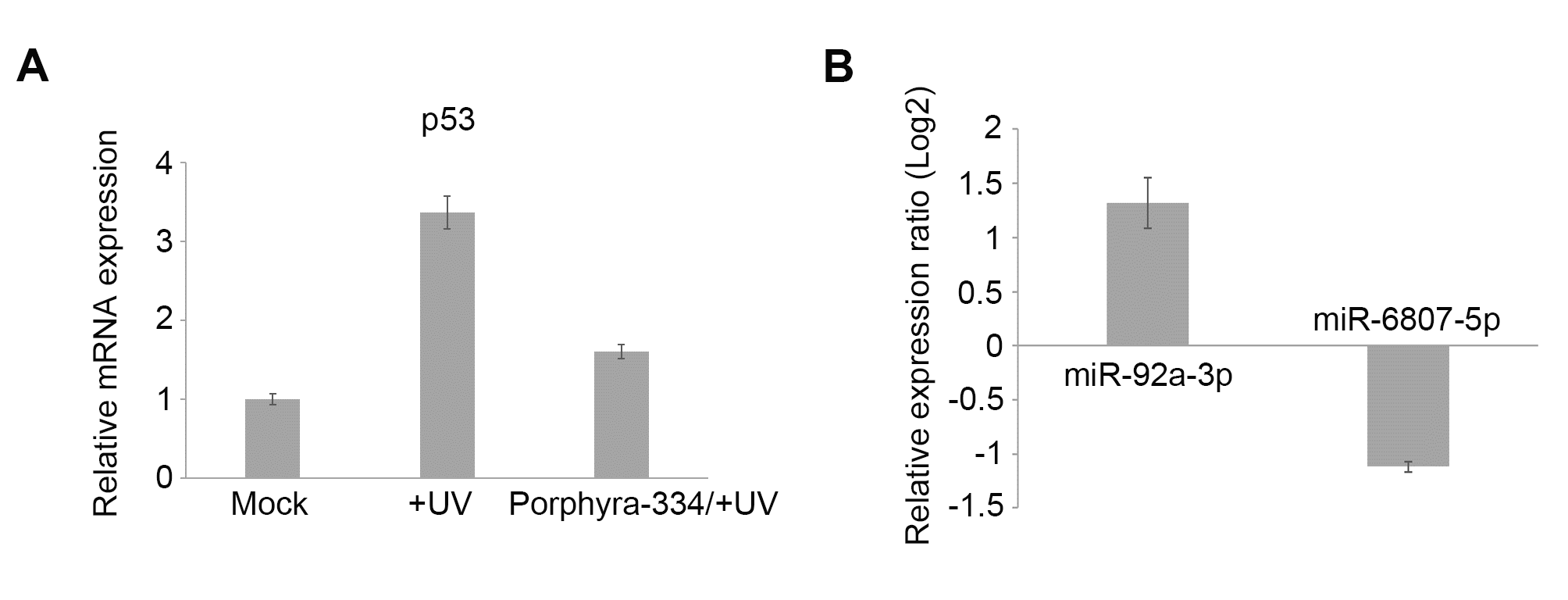

Supplement: Supplementary file 1 [file marinedrugs-15-00196-s001.zip › Supplementary Figure 3.tif]

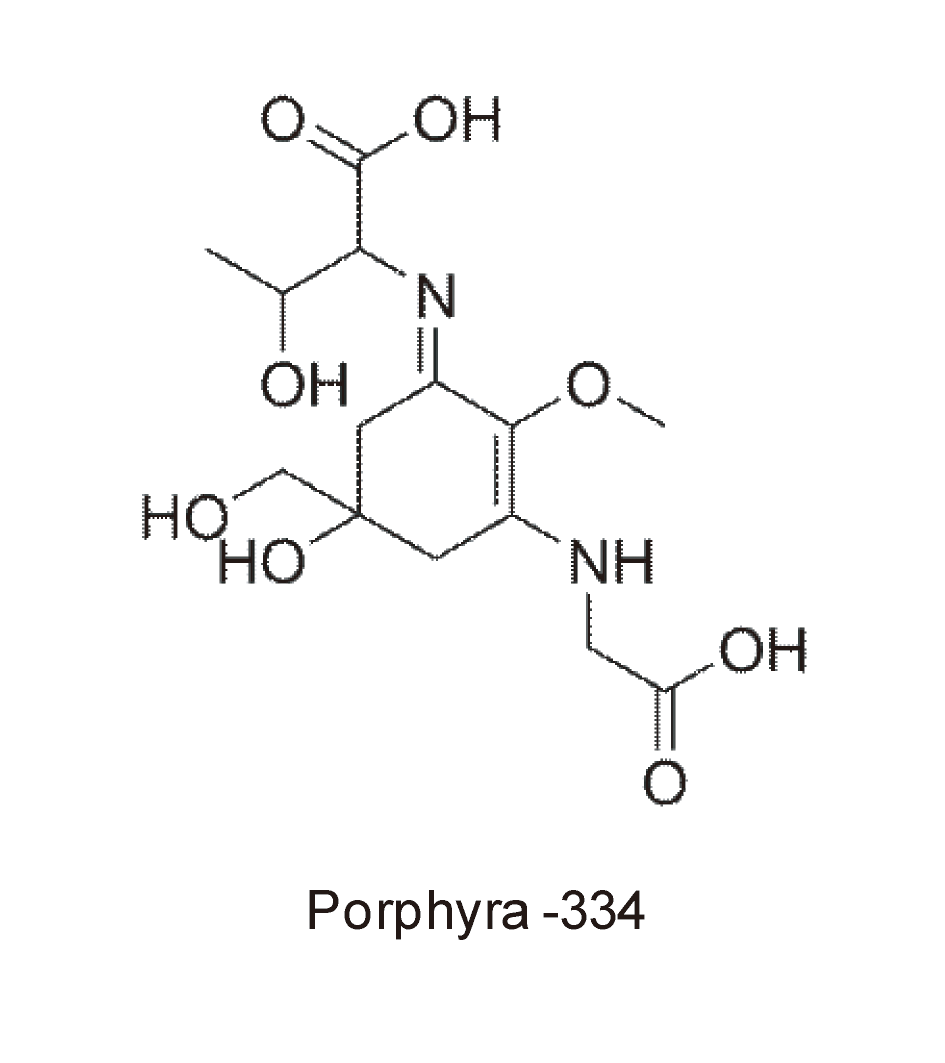

Supplement: Supplementary file 1 [file marinedrugs-15-00196-s001.zip › Supplementary Figure 1.tif]

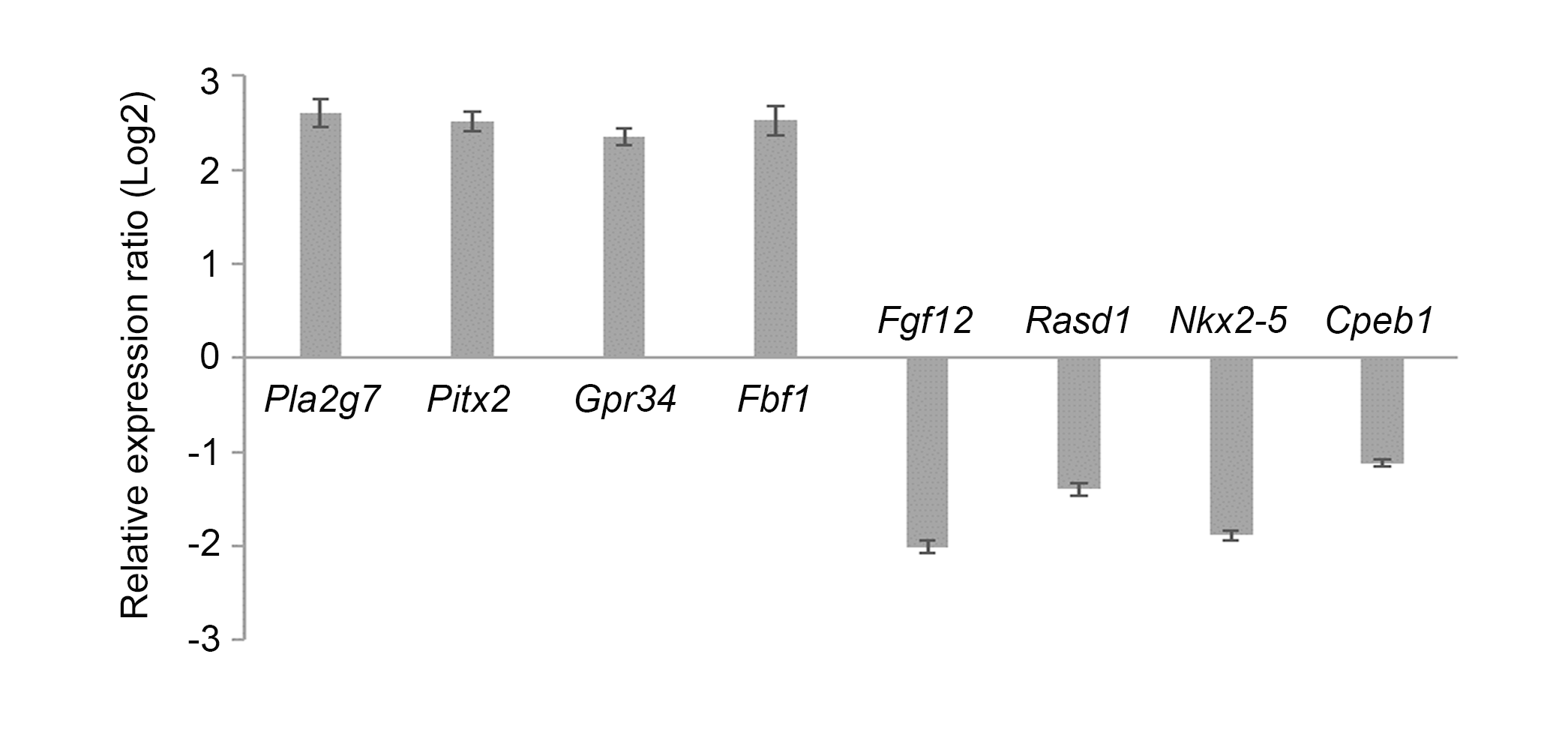

Supplement: Supplementary file 1 [file marinedrugs-15-00196-s001.zip › Supplementary Figure 2.tif]
